# *Nosema ceranae* Infections in Honey Bees (*Apis mellifera*) Treated with Pre/Probiotics and Impacts on Colonies in the Field

**DOI:** 10.3390/vetsci8060107

**Published:** 2021-06-10

**Authors:** Shane S. Klassen, William VanBlyderveen, Les Eccles, Paul G. Kelly, Daniel Borges, Paul H. Goodwin, Tatiana Petukhova, Qiang Wang, Ernesto Guzman-Novoa

**Affiliations:** 1 School of Environmental Sciences, University of Guelph, 50 Stone Road East, Guelph, ON N1G 2W1, Canada; smklassen@outlook.com (S.S.K.); wvanblyd@uoguelph.ca (W.V.); pgkelly@uoguelph.ca (P.G.K.); pgoodwin@uoguelph.ca (P.H.G.); 2 Ontario Beekeepers’ Association Technology Transfer Program, 185, 5420 Hwy 6 N, Orchard Park Office, Guelph, ON N1H 6J2, Canada; les.eccles@ontariobee.com (L.E.); dan.borges@ontariobee.com (D.B.); 3 Department of Population Medicine, University of Guelph, 50 Stone Road East, Guelph, ON N1G 2W1, Canada; tpetukho@uoguelph.ca; 4 Institute of Apicultural Research, Chinese Academy of Agricultural Sciences, 2 Yuanmingyuan W. Rd., Beijing 100193, China; beeprotect@yeah.net

**Keywords:** prebiotics, probiotics, *Nosema ceranae*, honey bee, longevity, bee populations, honey production, honey bee longevity, winter mortality

## Abstract

Alternatives to the antibiotic fumagillin for the control of *Nosema ceranae*, a gut parasite of the honey bee, are needed. The prebiotics eugenol, chitosan, and naringenin and the probiotic Protexin^®^ (*Enterococcus faecium*) provided in sugar syrup or protein patty either in spring or fall were evaluated for their effects on *N. ceranae* infection, colony population, honey yield and winter survivorship using field colonies. In the first year, spring treatments with eugenol, naringenin, and Protexin^®^ significantly reduced *N. ceranae* infection and increased honey production, while Protexin^®^ also increased adult bee populations and chitosan was ineffective. Fall treatments increased survivorship and decreased *N. ceranae* infection the following spring. In the second year, selected compounds were further tested with a larger number of colonies per treatment and only protein patty used in the spring and sugar syrup in the fall. Protexin^®^ and naringenin significantly decreased *N. ceranae* infections and increased the population of adult bees after spring treatment, but did not affect honey yields. There were no differences between treatments for colony winter mortality, but surviving colonies that had been treated with Protexin^®^ and naringenin were significantly more populated and had lower *N. ceranae* spore counts than control, non-treated colonies. Protexin^®^ and naringenin were the most promising candidates for controlling *N. ceranae* and promoting honey bee populations, warranting further investigation. Future research should investigate the optimal colony dose and treatment frequency to maximize colony health.

## 1. Introduction

*Nosema ceranae* and *N. apis* are obligate microsporidian parasites of the honey bee ventriculus, causing chronic infections [1]. The life cycle of *N. ceranae* and *N. apis* are similar with spores being ingested by young bees and then germinating in the ventriculus, penetrating epithelial cells, reproducing and rupturing host cells, and reentering the gut in as little as six days [2]. *N. ceranae* has replaced *N. apis* as the most prevalent microsporidian in honey bee colonies in Asia, Europe, and the Americas [3,4,5,6] and infection levels by *N. ceranae* can be significantly higher than those of *N. apis* [7]. The impact of *N. ceranae* on colonies can be severe due to bee depopulation, reduced brood rearing, slow spring development, reduced honey stores, and higher winter mortality [8,9,10,11,12].

For over 60 years, fumagillin has been the only registered antibiotic for *Nosema* spp. infections of honey bees in North America [13]. While effective, fumagillin poses concerns for food safety due to the contamination of hive products, such as honey and wax [14,15]. Additionally, the commercial formulation of fumagillin sold in North America was recently discontinued. One alternative to control *Nosema* spp. infections of honey bees could be feeding bees prebiotics and probiotics.

Prebiotics are non-digestible food ingredients that benefit animals by selectively stimulating the growth and activity of certain microbes in the digestive tract while sometimes inhibiting others [16]. For example, chitosan, a deacetylated form of chitin, is a prebiotic that can be directly antimicrobial, such as inhibition of the growth of *Serratia marcescens*, a bacterial pathogen of *Bombyx mori*, both in vitro and in vivo [17], and can also stimulate the honey bee immune system [18]. Administration of chitosan to honey bees resulted in a significant decrease in the number of *N. ceranae* spores and an increase in survivorship of the infected bees [19]. Many components of essential oils are prebiotics, such as eugenol from clove oil, that thickened the inner mucus layer of mice, increasing resistance to the enteric pathogen *Citrobacter rodentium* [20]. Flavones can also be prebiotics, such as naringenin from citrus fruits, that has anti-inflammatory properties increasing the abundance and activity of antioxidants, scavenging ROS and other free radicals, and decreasing the levels and activity of pro-inflammatory cytokines in mice [21]. Feeding it to caged honey bees in the laboratory resulted in a 64% reduction in *N. ceranae* spores with bees fed naringenin living as long as non-infected control bees and significantly longer than infected bees [22]. Other prebiotics have also shown promise for the control of *N. ceranae* infections in honey bees in laboratory studies. For example, Nanetti et al. [23] found that administration of defatted seed meals from *Brassica nigra* in the diet of honey bees inhibits *N. ceranae* replication and increases the lifespan of the insects. Part of the antimicrobial activity of these seed meals seems to be associated to the production of isothiocyanates in the gut of the bees. However, the previously mentioned prebiotics have not been tested in colonies in field studies.

Probiotics are living microbial food supplements that benefit animals by altering the balance of intestinal microorganisms [24]. One example is *Enterococcus faecium,* which is found in the intestines of a range of animals, including honey bees, although it is not one of their core gut bacteria [25]. Feeding *E. faecium*, for example, affected the intestinal microbial flora and the immune response of mice [26]. *E. faecium* also produces lactic acid in the midgut of the honey bee [25], which causes thickening of the peritrophic membrane in the ventriculus epithelium [27]. As *Nosema* must go through this membrane to infect epithelial cells, this may be a mode of defense against *Nosema* infection [28]. Moreover, addition of *E. faecium* to pollen substitutes increased digestibility by honey bees and reduced their mortality [29]. There are many commercially formulated products containing *E. faecium*, such as Protexin^®^, that contains a single strain of *E. faecium* marketed for improving the microbiota and gut health of farm animals. However, it also appears to function in honey bees as Borges et al. [30] found that treating honey bees with Protexin^®^ decreased *Nosema* spore numbers by >58% and significantly increased survival relative to both *N. ceranae*-inoculated and non-inoculated bees. However, this probiotic has not been tested in hives in field studies.

The objective of this study is to determine the effect of the prebiotics, eugenol, chitosan and naringenin, and the commercial probiotic Protexin^®^ (*E. faecium*) on *N. ceranae* infections, bee populations, honey production, and overwintering survival of honey bee colonies. The hypothesis is that feeding pre/probiotics to honey bees infected with *N. ceranae* would result in a significant reduction in *N. ceranae* infection and increased colony populations, honey yields, and over-wintering survival.

## 2. Materials and Methods

### 2.1. Colony Selection and Management

The experimental setup consisted of two trials conducted in consecutive years. The 2017 trials were intended to test three prebiotics and one probiotic with two different delivery methods during spring and fall. The 2018 trials were intended to test the two most effective prebiotic/probiotics and the most efficient delivery methods from the 2017 trials, using a larger sample size per treatment to validate results from 2017.

In 2017, over 150 honey bee colonies from the University of Guelph’s Honey Bee Research Centre in Ontario, Canada, were screened for *Nosema* spp. spores in early April. To collect older bees, which consistently show the highest *Nosema* infection levels [27,31], approximately 100 bees were obtained from the inner cover of each colony. They were stored in jars containing 100 mL of 75% ethanol and assayed for *Nosema* spore presence and numbers, as described below. The *Nosema* species in the infected colonies was determined by species-specific PCR [32], and all were *N. ceranae*. Following screening, 6 positive control, 6 negative control, 6 standard control, and 6 colonies per each of eight pre/probitoic treatments were moved to two apiaries that were <1 km apart from each other and that had similar bee forage. To reduce apiary location effects, colonies from each treatment group were evenly divided between the two locations (3 colonies at each location per treatment). All colonies were sampled again for *N. ceranae* spores in early May prior to treatments as per early spring and analyzed for *N. ceranae* spores as described below. Based on spore numbers, the colonies were evenly distributed among the treatments so that each had a mean number of *N. ceranae* spores per bee of 3.88 × 10^6^ ± 4.80 × 10^5^. Negative control colonies treated with fumagillin in the previous year were also examined to determine that no spores were present. Colonies were individually identified by a numbered livestock tag identifying their treatment.

In 2018, over 200 colonies from commercial apiaries were screened for *N. ceranae* in early April as in 2017, and 100 colonies were selected. Again, screening with a species-specific PCR assay [32] showed that all the selected colonies were only infected with *N. ceranae*. The 100 colonies were established in three apiaries located at <1 km from each other in Fergus, ON, Canada. All colonies were sampled again for *N. ceranae* spores in early May as in 2017 prior to treatment, and the 33 positive control and 33 or 34 colonies per pre/probitoic treatment were evenly distributed among the three locations. All colonies were treated with Apivar^®^ (Amitraz; Véto-Pharma, Paris, France) as per the label to control *Varroa destructor* infestations. To prevent swarming that would affect bee populations and honey production, all frames were removed from the colonies and swarm cells were destroyed when detected. Colonies were hibernated over winter outdoors.

### 2.2. Treatments

Colonies in 2017 were treated with eugenol (Sigma-Aldrich, Oakville, ON, Canada), naringenin (Sigma-Aldrich, Oakville, ON, Canada), chitosan (DNP, Quebec City, QC, Canada), and Protexin^®^ (Probiotics International, Lopen, Somerset, UK). Prebiotics used are technical grade (>98% purity) and Protexin^®^ contains *E. faecium* (2 × 10^12^ CFU/kg). The dose per bee of each compound was estimated from data of previous studies [19,22,30]. The dose per hive was calculated using the estimated dose per bee, which was multiplied by 12,000, that is the estimated number of individuals in the spring in a typical honey bee colony in a northern temperate region [33]. The positive control consisted of colonies infected with *N. ceranae* receiving no treatment, the negative control consisted of *Nosema*-negative colonies receiving no treatment, and the standard control consisted of colonies infected with *N. ceranae* and receiving the antibiotic fumagillin as per the label (Medivet Pharmaceuticals Ltd., Edmonton ON, Canada).

Treatments were administered to each colony in either 2 L sucrose syrup (S) containing 50% sucrose, or a 0.45 Kg protein patty (P) composed of 300 g sucrose, 76 g brewer’s yeast (Bulk Barn, Guelph ON, Canada), 36 g egg powder (Elmira Pet Products, Elmira, ON, Canada), 33 mL dH20, and 5 mL canola oil (personal communication, Ontario Beekeepers Association Technology Transfer Program). Fumagillin was applied in sucrose syrup. Syrup or patties without treatments were administered to the positive and negative control colonies. All colonies with syrup treatments also received a non-treated protein patty, and all colonies with patty treatments also received 2 L of non-treated syrup. Thus, all colonies received 2 L of sucrose syrup and one protein patty with or without treatment. Patties were sandwiched in a sheet of wax paper and placed on the top bars of the colonies above the brood nest, and syrup was delivered to the colony in hive-top feeders. Spring and fall treatments took place on 31 May and 28 September, respectively. Spring treatments were applied before honey supers were added to the colonies to prevent potential contamination of honey. Fall treatments were applied after honey supers were harvested for the same reason.

Colony treatments in 2018 were a *N. ceranae* positive control (*n* = 33), Protexin^®^ (*n* = 34), and naringenin (*n* = 33). Treatments were applied in spring in pollen patties and in fall in sucrose syrup at the doses used in 2017. As in 2017, all colonies received 2 L of sucrose syrup and one protein patty with or without treatment. Negative and fumagillin control groups were not included, as they had been examined in 2017. Spring and fall treatments took place on 31 May and 28 September, respectively.

### 2.3. Nosema ceranae Sample Collection and Quantification

To detect changes in *N. ceranae* spore numbers, samples of approximately 200 adult worker bees were collected from the inner cover of each colony and stored in 100 mL 75% ethanol at one to two days before treatment (pre-treatment) and three weeks after the spring or fall treatment (post-treatment) [34]. Samples were also collected the following spring.

The abdomens of 100 bees from each sample were placed in dH20 (1 mL/abdomen) in 7” × 11” 3 mm plastic vacuum bags (Shortreed, Guelph, ON, Canada) and then macerated for 60 s in a Stomacher machine (Stomoacher 400 Circulator, Seward, Worthing, West Sussex, UK) [35]. Then, 10 μL of the homogenate was placed on a slide and seen under a phase contrast microscope at 400× (Omax 40×–2500×, United Scope Inc., Kitchener, ON, Canada) to look for *Nosema* spp. spores. When detected, a hemacytometer was loaded with homogenate, and the spores quantified [36]. The intensity of *N. ceranae* infections can be reliably quantified by microscopy spore counts because they correlate with PCR quantification methods (R^2^ = 0.95) [32].

### 2.4. Colony Populations and Overwintering Survival

To determine the effects of treatments on colony population growth, colonies were assessed for spring treatments at one to two days before treatment and then nine weeks later as per Delaplane et al. [37]. Colonies were also assessed the following spring. During a colony assessment, all combs in a colony were removed, and the adult population was determined by counting the number of combs covered with adult bees, and the brood population was calculated by counting the number of frames containing brood. Overwintering colony survival rate was determined by counting the number of colonies containing live queens and workers in May of 2018 and May of 2019.

### 2.5. Honey Production

Honey supers were harvested from colonies on 11 August and 28 September in both 2017 and 2018. Supers were labeled with their hive number. The supers were individually weighed before and after honey extraction using a single beam platform scale (Detecto 500 lb, Cardinal Scale Manufacturing, Webb City, MI, USA). Care was taken to return the frames to their original super ensuring that any weight differences could be attributed only to honey yield.

### 2.6. Statistical Analyses

The assumption of normality for the data of *Nosema* spore numbers, frames with brood or bees and honey yields were verified with the Shapiro–Wilk test, and the homogeneity of variances in the treatments was determined with the Bartlett test. Because the data of several variables were not normally distributed and their variances were heterogeneous, the data on spore numbers were log transformed, and the data on number of frames with brood or adult bees were subjected to a power of—1 transformation to normalize them. Honey yield data did not require transformation. The associations between the treatments and the estimated response difference in spore numbers between pre and post-treatment were examined with a generalized estimating equation (GEE) model. The response variable is spore numbers, which was modeled with a Poisson distribution in the analysis, because it takes into account the over dispersion of spore counts and the dependence of the spore counts within colonies. The associations between the treatments and the difference in number of frames with brood or adult bees between pre and post-treatment were analyzed with a linear regression model. For the honey yield data, comparisons were made between *N. ceranae* positive and negative control colonies, as well as between positive control colonies and the different treatments using Student t-tests. Percent colony overwintering survival was examined with Fisher exact tests only in 2019. The overwintering survival data of 2018 were not statistically analyzed because of the low number of surviving colonies of some treatments. Data on *N. ceranae* spore numbers, adult bee, and brood frames from overwintered colonies were subjected to analyses of variance after transforming them as explained above. All statistical analyses were performed with a significance level of *p* < 0.05 using R, version 3.3.1 (R Foundation for Statistical Computing, Vienna, Austria).

## 3. Results

### 3.1. Spring 2017 Treatments

In spring 2017, bees from positive control colonies showed *N. ceranae* infection at the time of treatment, which declined by three weeks, whereas bees from negative control colonies were associated with a significant increase in *N. ceranae* spores per bee (*p* < 0.01) over the same period (Table 1). Spring treatment with eugenol P, Protexin^®^ P, and naringenin S significantly decreased the number of spores per bee compared to the positive control after three weeks (*p* < 0.05). These treatments all reduced spore counts by approx. 89%. Neither fumagillin nor any of the other treatments had significant effects on spore counts by three weeks following spring treatments.

**Figure 1:**
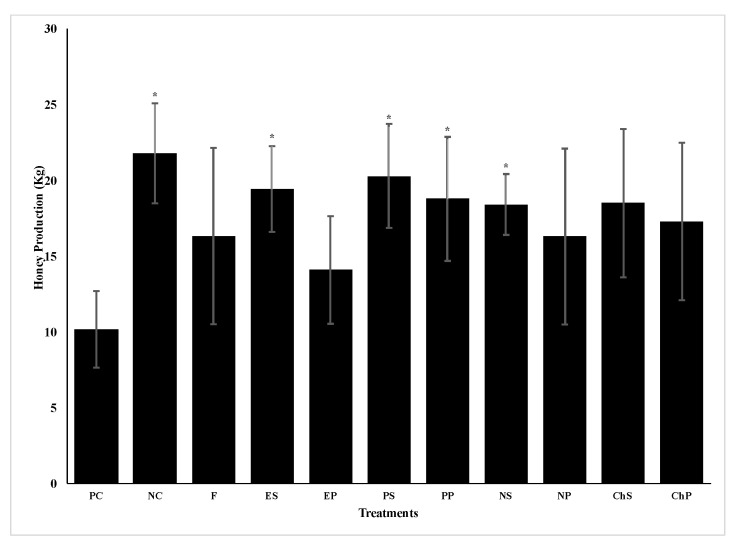
Effect of spring 2017 treatments on honey production per colony (Kg ± SE) three months post-treatment for positive control (PC), negative control (NC), fumagillin (F) applied in syrup, eugenol in syrup (ES), eugenol in patty (EP), Protexin^®^ in syrup (PS), Protexin^®^ in patty (PP), naringenin in syrup (NS), naringenin in patty (NP), chitosan in syrup (ChS) and chitosan in patty (ChP). Means are from six colonies per treatment. * indicate significant differences (*p* < 0.05) relative to the PC based on t tests.

Positive and negative control colonies were not significantly different in the change of number of brood frames nine weeks after spring treatments (Table 2). Although several spring treatments significantly reduced the number of spores per bee by three weeks (Table 1), none of the treatments significantly increased the number of brood frames by nine weeks after treatment (Table 2). Only chitosan S treatment had an effect, which was to significantly decrease brood frames compared to the positive control (*p* < 0.05). Like brood frames, the change in number of frames covered with adult bees for positive and negative control colonies nine weeks after the treatments were not significantly different (Table 3). Only Protexin^®^ P treatment resulted in a significant increase in frames with adult bees by nine weeks after spring treatment compared to the positive control (*p* < 0.05). While an increase in frames with adult bees was similar with eugenol S spring treatment, the change was not significant. Positive control colonies yielded significantly less honey (10.2 ± 2.2 kg) than the negative control colonies (21.8 ± 3.3 Kg) (t = 2.90, *p* < 0.05) by nine weeks after the time of treatments. Among the treatments, only eugenol S, Protexin^®^ S, Protexin^®^ P, and naringenin S spring-treated colonies had significantly higher honey yields compared to colonies of the positive control (t = 2.55, t = 2.33, t = 2.26, and t = 2.31, *p* < 0.05, respectively; Figure 1).

### 3.2. Fall 2017 Treatments

Bees of positive and negative control colonies both showed infection at the time of treatment, and the number of *N. ceranae* spores per bee increased by 130 and 405%, respectively, over three weeks during the fall (Table 4). However, the changes were not significant. Fall treatments with only Protexin^®^ S and fumagillin significantly decreased the number of spores per bee in three weeks compared to the positive control by 75% (*p* < 0.01) and 69% (*p* < 0.05), respectively (Table 4). By the spring of 2018, the lowest *N. ceranae* spore numbers per bee compared to the positive control colonies were for colonies that were treated the previous fall with fumagillin at 55 times lower, chitosan P at 12.9 times lower, and Protexin^®^ S or naringenin S at 6.4 times lower, although none of the differences were significant (F_10,33_ = 1.27, *p* = 0.28; Table 5). Moreover, the overwintered colonies in spring 2018 had the highest survival rate with Protexin^®^ P, Protexin^®^ S, and naringenin P fall treatments, followed by eugenol S and eugenol P fall treatments (Table 5), although no statistical comparison was performed due to low colony numbers. The number of frames containing brood in the spring was highest with fall treatments of chitosan P, Protexin^®^ P, and eugenol P, which were 85% or more above that of the positive control, but none of the differences were significant (F_10,33_ = 0.48, *p* > 0.05). Although colonies that had been treated with fumagillin, eugenol P and Protexin^®^ S the previous fall had two times as many frames covered by adult bees compared with positive control colonies in the spring, those differences also were not significant (F_10,33_ = 0.66, *p* > 0.05). In general, there was considerable variability in the results for overwintering bees, particularly for some treatments, like chitosan P, indicating the need to compare a greater number of colonies in the following year.

### 3.3. Spring 2018 Treatments

Selected treatments were repeated with a larger sample size of 33–34 colonies per treatment in spring 2018 versus 6 colonies per treatment in 2017. The retested spring treatments were Protexin^®^ P and naringenin P as protein patties were readily consumed within three weeks in spring but not all sugar syrup was consumed. In addition, Protexin^®^ P spring 2017 treatment resulted in significant reduction in *N. ceranae* spore numbers and increased number of frames covered with adult bees, while naringenin P spring 2017 treatment resulted in a similar, but not significant, reduction in spore numbers (Table 1 and Table 4). In 2018, both Protexin^®^ P and naringenin P spring treatments were significantly associated with reduced *N. ceranae* spore numbers compared to the positive control three weeks after treatment (*p* < 0.05; Table 6). Neither Protexin^®^ P nor naringenin P spring treatments significantly altered the number of brood frames nine weeks after treatment (Table 7), but both treatments were associated with a significant increase in the number of frames covered with adult bees compared to the positive control (*p* < 0.05; Table 8). Positive control, Protexin^®^ P and naringenin P spring treatments yielded 47.2 ± 3.5, 49.5 ± 4.0, and 52.0 ± 4.2 Kg of honey per colony nine weeks after treatment, respectively, which were not significantly different (F_2,97_ = 0.379, *p* = 0.68).

### 3.4. Fall 2018 Treatments

The treatments repeated in fall 2018 with the larger colony sample size were Protexin^®^ S and naringenin S as sugar syrup could be consumed completely in the colonies within three weeks prior to winter storage but not all the patties. Moreover, Protexin^®^ S fall 2017 treatment significantly reduced the spore numbers three weeks later as well as increased overwintering colony survival, and naringenin S fall 2017 treatment reduced spore numbers the following spring (Table 2 and Table 5). Protexin^®^ S and naringenin S fall 2018 treatments did not result in reduced spore numbers per bee three weeks after treatment compared to the positive control (Table 9). However, by the spring of 2019, both the Protexin^®^ P and naringenin P fall treatments resulted in significantly lower spore numbers per bee than those of positive control colonies, but were not significantly different from each other (F_2,69_ = 5.01, *p* < 0.01; Table 10). Furthermore, the overwintered colonies in spring 2019 showed that the highest survival rate (79.4%) was for colonies treated with Protexin^®^ P during fall, which was approx. 13% higher than that of the positive control colonies (66.6%), but none of the differences were significant. The overwintered colonies in spring 2019 showed no significant effect of fall treatment on the number of brood frames (F_2,69_ =1.89, *p* > 0.05), but both Protexin^®^ P and naringenin P fall treatments resulted in significantly more frames covered with adult bees in spring 2019 than positive control colonies (F_2,69_ = 3.83, *p* < 0.05; Table 10).

## 4. Discussion

Analyses of the positive and negative control colonies in spring 2017 showed that while the negative control colonies started the experiment without infection by *N. ceranae*, they were rapidly infected when placed in an apiary next to infected colonies. This was expected as Higes et al. [34] also found that *N. ceranae* negative colonies became rapidly infected after being placed adjacent to infected hives due to bee drift or spore movement by the wind [38]. Thus, negative control colonies contained spores at the time of the fall 2017 treatments as well as in the following spring. However, despite becoming infected, colonies of the negative control produced twice as much honey as colonies of the positive control that had high *N. ceranae* infection levels at the beginning of the study. Other studies of field colonies infected with different levels of *N. ceranae* infection also found significant reductions in honey yield in highly infected colonies [8,12]. *Nosema ceranae* infection can reduce honey yields because infected bees have impaired nutrient absorption and the pathogen uses large amounts of energy from their hosts, resulting in the bees having significantly less energy for activities such as foraging behavior [39]. Infection may also result in smaller colonies [12,19,39]. While some studies have shown that *N. ceranae* infections reduce the lifespan of bees [12,19], other studies have reported no significant reduction in lifespan due to infection [40,41]. This study also indicates no reduction in lifespan as colony populations were comparable based on a lack of significant differences in the number of frames with brood or adult bees between negative and positive control colonies in late spring 2017. As honey production is a highly variable trait (CV >65%) [42] affected by many environmental factors, an experiment with a much larger sample size is necessary to confirm the effect of *N. ceranae* infections on colony honey yields. However, due to the limited number of colonies with the greater replicates in 2018, no negative control colonies were included in the second year of this study.

Fumagillin was included in this study as a standard control treatment for the 2017 experiment. However, this treatment was not associated with a significant decrease in *N. ceranae* spores after the spring 2017 treatment. This suggests that fumagillin was ineffective in bees with an established infection, which shows that the antibiotic on which North American beekeepers once relied can be ineffective when applied as per the label for spring treatment. A study by Huang et al. [13] showed that *N. ceranae* infections were not affected by treating honey bees with fumagillin, which agrees with our results. Spring fumagillin treatment also did not increase the number of frames with brood or adult bees, nor honey yield compared to the positive control. In contrast, fall fumagillin treatment significantly decreased the number of *N. ceranae* spores per bee three weeks later, and a large but non-significant decrease the following spring, suggesting there may be a factor of seasonality in the efficacy of this antibiotic. Considering its variable effectiveness in this study as well as the health risks of fumagillin, such as antibiotic resistance and contamination of honey and wax [15], it is clear that alternatives to this antibiotic are needed than can match or exceed its effectiveness.

Compared to the pre-treatment levels, spring 2017 eugenol P treatment was associated with a reduction of spores per bee by >89% and fall 2017 eugenol S treatment reduced spores per bee by >45% three weeks later (although the spore numbers were slightly higher by the following spring), thus demonstrating its pathogen control effectiveness in this study. Eugenol in sugar syrup also decreased *N. ceranae* spore numbers in caged bees [30]. Eugenol may have reduced *N. ceranae* spore numbers in bees by interfering with enzymes involved in fungal spore germination, such as it does against *Aspergillus* species [43], or it may have reduced the chance of *N. ceranae* infection by thickening the inner layer of the intestinal tract, such as it does in mice increasing resistance to the enteric pathogen *Citrobacter rodentium* [20]. As *N. ceranae* spores must penetrate this layer membrane to infect epithelial cells, thickening it might explain part of the results seen in this study. Further investigation could reveal if eugenol has similar effects on *N. ceranae* spore germination or the thickness of the midgut lining of the honey bee. Despite reduced spore numbers per bee, none of the spring or fall eugenol treatments significantly affected brood or adult bee populations. However, spring eugenol S treatment significantly increased honey production compared to the positive control. Similar to the difference between the positive and negative control colonies, honey production may have increased as reduced infection in eugenol treated bees may have allowed for greater foraging.

Among the treatments in this study, Protexin^®^ P was most effective in the spring 2017 treatment in reducing *N. ceranae* spore numbers, whereas Protexin^®^ S was the most effective for fall 2017 treatment three weeks later, as well as resulting in one of the lowest spore numbers by the following spring among the treatments. Thus, Protexin^®^ was also tested for a second year. Protexin^®^ P spring treatment was also effective against *N. ceranae* in 2018, although the Protexin^®^ S fall treatment was only effective in reducing spore numbers the following spring and not the fall. Thus, Protexin^®^ was more consistent in its ability to control *N. ceranae* infection in the field than fumagillin, the only registered product for control. Similarly, including it in the sugar syrup of caged honey bees reduced *N. ceranae* infection [30,44]. This could be related to the ability of *E. faecium* to produce antimicrobial bacteriocin-like compounds [25] that might aid in the prevention of *N. ceranae* infection in bees. *E. faecium* also produces lactic acid in the gut of honey bees [25], which has been shown to reduce *N. ceranae* infection when produced by other microorganisms in the digestive tube of honey bees [45,46]. Additionally, lactic acid can cause thickening of the peritrophic membrane in the gut epithelium of honey bees [47]. A thicker membrane could reduce the chances of *N. ceranae* spores infecting epithelial cells of honey bees. However, it may have other benefits to the bee. Feeding caged honey bees *E. faecium* resulted in increased life span of bees [30]. This may help explain why, in both years of this study, Protexin^®^ P spring treatment increased the number of adult bees in colonies, and Protexin^®^ S fall 2018 treatment increased adult bee populations that survived over winter compared to the positive control. However, Protexin^®^ P did not increase the number of brood frames, although Protexin^®^ S fall treatment did for overwintering colonies in both years of the study, but only significantly in the second year. This implies that Protexin^®^ is likely extending the life span of the bees, rather than allowing the colony to rear more bees, except in the overwintered bees. This result is consistent with the increased life span of bees treated with Protexin^®^ reported by Borges et al. [30]. Regardless of the impact of *N. ceranae* infection on bee life span, Protexin^®^ not only reduced *N. ceranae* proliferation, but increased bee survivorship compared to infected and non-infected bees [30], which appeared to occur in this study. Additionally, colonies treated with Protexin^®^ S and Protexin^®^ P produced significantly more honey than positive control colonies in 2017, with production similar to that of a healthy colony. While this could be due to the increased adult bee population, Protexin^®^ treatment did not increase honey production in 2018, even though it also increased adult bee populations. Honey yields can be quite variable making it difficult to find treatment effects. For example, Fanciotti et al. [48] found that colonies of honey bees fed *Lactobacillus salivarius* yielded significantly more honey than the control in one year, but not in a subsequent year. Larger numbers of colonies over many years of study will be required to confirm the effect of probiotics on honey yields. Overall, Protexin^®^ treatment of honey bee colonies in this study was consistently associated with the most positive effects on bees, making it the most successful compound at minimizing the damaging effects of *N. ceranae.*

Naringenin S spring 2017 treatment significantly reduced *N. ceranae* infection, whereas naringenin S fall 2017 treatment had a nearly significant reduction in infection in the following spring. Thus, it was tested again in the subsequent year using more colonies, showing a significant effect with both naringenin P spring 2018 treatment at three weeks later and naringenin S fall 2018 treatment in the following spring. Naringenin treatment in sugar syrup similarly reduced *N. ceranae* spore numbers in caged honey bees [22]. Reduced multiplication of *N. ceranae* with naringenin could be due to its anti-inflammatory activity that increases the abundance of antioxidants and decreases the levels of pro-inflammatory cytokines, thus increasing host resistance [21]. Although spring treatment with naringenin was not associated with larger adult or brood bee populations in 2017, it was significantly related with more adult bees in the larger-scale experiment in 2018. Honey production changes with naringenin treatment group were significantly higher in 2017 but not 2018, which could reflect the high variability in this parameter as mentioned previously with Protexin^®^.

Chitosan was never associated with a significant reduction in spore numbers in this study, although chitosan P fall 2017 treatment resulted in a large reduction in spore numbers the following spring. Chitosan can act as a pathogen-associated molecular pattern, binding to pattern recognition receptors to trigger an immune response against pathogens [49]. The ineffectiveness of chitosan in this study was surprising as studies had found that feeding chitosan in sugar syrup reduced *N. ceranae* infections in caged honey bees [19,22] and also decreased infections of *N. apis* in a laboratory study [18]. Chitosan S spring 2017 treatment did not increase the amount of adult bees and actually reduced the amount of brood, while chitosan fall 2017 treatment gave highly variable results. Borges et al. [22] did not find higher adult bee survival with chitosan treatment, whereas both Saltykova et al. [18] and Valizadeh et al. [19] reported higher survival with chitosan. Perhaps the positive effects observed in studies in the laboratory are not reflective of what would occur in the field. A possible explanation for the negative effect of chitosan on brood number is that it may have initiated an immune response causing the bees to use excess energy and thus decrease temporarily the energy for rearing brood.

In this study, all the pre/probiotics were administered to honey bee colonies by either incorporating them into sucrose syrup or a protein patty in 2017. This was done to determine if that could impact their effectiveness. The pattern seen across most treatments was that compounds administered in the protein patty worked better in the spring, whereas the sugar syrup worked better in the fall. Thus, only selected compounds in protein patty in the spring and sugar syrup in the fall were tested in 2018. The benefit of patties in spring could be related to the rapid consumption of the treated syrup compared to the patty, thus limiting the period of treatment. Corby-Harris et al. [50] found that microbes reproduced well and were efficiently distributed within a hive when administered in pollen patties during spring. The benefit of syrup in fall could be related to the slow patty consumption by bees in the fall, with some colonies never finishing their patties prior to overwintering, whereas the syrup was consistently completely consumed prior to overwintering providing full treatment. Therefore, the most effective administration method may be due to feeding compounds in a form that the colony will completely but not too rapidly consume based on the time of year (patties in the spring and syrup in the fall).

## 5. Conclusions

Based on this study, Protexin^®^ and naringenin seem to be good candidates for mitigating *N. ceranae* infections, as well as for promoting length of life and possibly productivity in honey bee colonies. The decrease in *N. ceranae* spores may be the reason for Protexin^®^ and naringenin increasing adult bee populations, presumably due to increased longevity. Therefore, these compounds warrant further investigation. Another area of future research could be to determine optimal dosages and administration frequency, as the colonies may benefit from more frequent treatments. As they may also have positive effects on general bee health, both compounds could also be tested in healthy colonies to determine if they can increase bee populations and performance.

## Figures and Tables

**Table 1:** Effect of spring 2017 treatments on *Nosema ceranae* spore numbers per bee one-two days pre-treatment and three weeks post-treatment with the antibiotic fumagillin, prebiotics eugenol, naringenin or chitosan, or probiotic Protexin^®^. Coefficients indicate how the log of spores per bee changed relative to the positive control. Means are from 100 bees in six colonies for each treatment.

Treatment ^1^	Spores/Bee	Spores/Bee	% Change	Coefficient ^2^
	Pre-Treatment	Post-Treatment	Spores/Bee	
	(Millions) ± SE	(Millions) ± SE		
Positive Control	5.10 ± 0.96	2.68 ± 0.56	−47.4	−0.65
Negative Control	0.00 ± 0.00	0.54 ± 0.09	54	5.34 **
Fumagillin S	4.22 ± 0.64	2.00 ± 0.54	−52.6	−0.1
Eugenol S	2.85 ± 0.40	1.36 ± 0.53	−52.3	−0.09
Eugenol P	5.39 ± 1.06	0.55 ± 0.11	−89.8	−1.64 *
Protexin^®^ S	4.98 ± 0.87	1.56 ± 0.40	−68.7	−0.52
Protexin^®^ P	4.19 ± 0.54	0.45 ± 0.13	−89.3	−1.59 *
Naringenin S	4.06 ± 0.49	0.42 ± 0.07	−89.7	−1.63 *
Naringenin P	3.88 ± 0.45	0.45 ± 0.09	−88.4	−1.57
Chitosan S	3.87 ± 0.49	1.02 ± 0.33	−73.6	−0.69
Chitosan P	3.93 ± 0.52	0.84 ± 0.18	−78.6	−0.9

^1^ Treatments applied in a protein patty (P) or sucrose syrup (S). In spring 2017, non-treated positive control and treated colonies started with natural *N. ceranae* infection, and non-treated negative control colonies started with no detectable *N. ceranae*. ^2^ Significance at *p* < 0.05 is indicated by * and *p* < 0.01 is indicated by **.

**Table 2:** Effect of spring 2017 treatments on number of frames containing brood per colony one–two days pre-treatment and nine weeks post-treatment with the antibiotic fumagillin, prebiotics eugenol, naringenin or chitosan, or probiotic Protexin^®^. Coefficients indicate how the number of frames with brood changed relative to the positive control. Means are from six colonies per treatment.

Treatment ^1^	Frames w/Brood	Frames w/Brood	% Change	Coefficient ^2^
	Pre-Treat. ± SE	Post-Treat. ± SE	No. Frames	
Positive Control	8.88 ± 0.76	10.57 ± 0.54	19	−1.46
Negative Control	12.12 ± 0.24	10.54 ± 0.29	−13	−1.58
Fumagillin S	8.21 ± 0.99	8.67 ± 0.71	5.6	4.58
Eugenol S	8.65 ± 0.82	10.05 ± 0.20	16.2	1.47
Eugenol P	11.25 ± 0.48	8.59 ± 0.65	−23.6	1.07
Protexin^®^ S	8.58 ± 0.69	9.46 ± 0.45	10.3	1.44
Protexin^®^ P	9.91 ± 0.69	11.38 ± 0.22	14.8	1.34
Naringenin S	10.95 ± 0.24	8.88 ± 0.36	−18.9	−2.07
Naringenin P	10.12 ± 0.49	8.75 ± 0.83	−13.5	1.88
Chitosan S	12.01 ± 0.26	9.23 ± 0.10	−23.1	−2.75 *
Chitosan P	10.25 ± 0.89	11.79 ± 0.36	15	1.02

^1^ Treatments applied in a protein patty (P) or sucrose syrup (S). I spring 2017, non-treated positive control and treated colonies started with natural *N. ceranae* infection, and non-treated negative control colonies started with no detectable *N. ceranae*. ^2^ Significance at *p* < 0.05 is indicated by *.

**Table 3:** Effect of spring 2017 treatments on number of frames covered with adult bees per colony one–two days pre-treatment and nine weeks post-treatment with the antibiotic fumagillin, prebiotics eugenol, naringenin or chitosan, or probiotic Protexin^®^. Coefficients indicate how the number of frames with adult bees changed relative to the positive control. Means are from six colonies per treatment.

Treatment ^1^	Frames w/Adults	Frames w/Adults	% Change	Coefficient ^2^
	Pre-Treat. ± SE	Post-Treat. ± SE	no. Frames	
Positive Control	15.04 ± 1.66	18.83 ± 1.36	25.2	3.79
Negative Control	13.75 ± 1.74	16.58 ± 2.30	20.6	0.2
Fumagillin S	13.70 ± 3.19	18.58 ± 4.25	35.6	1.08
Eugenol S	10.00 ± 3.01	21.25 ± 4.89	112.5	6
Eugenol P	16.37 ± 3.85	21.58 ± 3.02	31.8	1.41
Protexin^®^ S	12.12 ± 1.70	19.54 ± 2.17	61.2	3.62
Protexin^®^ P	11.29 ± 1.75	23.87 ± 1.45	111.5	8.79 *
Naringenin S	12.91 ± 2.59	21.51 ± 2.99	66.6	4.79
Naringenin P	13.91 ± 2.18	20.00 ± 3.67	43.8	2.29
Chitosan S	14.62 ± 1.55	21.70 ± 2.51	48.4	3.29
Chitosan P	14.75 ± 1.81	22.25 ± 2.99	50.8	3.7

^1^ Treatments applied in protein patty (P) or sucrose syrup (S). In spring 2017, non-treated positive control and treated colonies started with natural *N. ceranae* infection, and non-treated negative control colonies started with no detectable *N. ceranae*. ^2^ Significance at *p* < 0.05 is indicated by *.

**Table 4:** Effect of fall 2017 treatments on *N. ceranae* spore counts per bee one-two days pre-treatment and three weeks post-treatment with the antibiotic fumagillin, prebiotics eugenol, naringenin or chitosan, or probiotic Protexin^®^. Coefficients indicate how the log of spores per bee changed relative to the positive control. Means are from 100 bees in six colonies per treatment.

Treatment ^1^	Spores/Bee	Spores/Bee	% Change	Coefficient ^2^
	Pre-Treatment	Post-Treatment	Spores/Bee	
	(Millions) ± SE	(Millions) ± SE		
Positive Control	0.82 ± 0.09	1.89 ± 0.44	130.5	0.84
Negative Control	0.57 ± 0.10	2.88 ± 0.73	405.3	0.79
Fumagillin S	0.70 ± 0.14	0.22 ± 0.05	−68.6	1.83 *
Eugenol S	1.73 ± 0.39	0.94 ± 0.19	−45.7	−1.45
Eugenol P	0.95 ± 0.18	2.79 ± 0.76	193.7	0.42
Protexin^®^ S	2.87 ± 0.46	0.72 ± 0.14	−74.9	−2.23 **
Protexin^®^ P	1.97 ± 0.20	1.88 ± 0.28	−4.6	−0.88
Naringenin S	0.88 ± 0.16	2.06 ± 0.23	134.1	0.01
Naringenin P	2.23 ± 0.36	2.11 ± 0.81	−5.4	−0.71
Chitosan S	0.81 ± 0.09	1.86 ± 0.16	129.6	−0.01
Chitosan P	1.27 ± 0.22	1.98 ± 0.30	55.9	−0.39

^1^ Treatments applied in a protein patty (P) or sucrose syrup (S). In spring 2017, non-treated positive control and treated colonies started with natural *N. ceranae* infection, and non-treated negative control colonies started with no detectable *N. ceranae*. ^2^ Significance at *p* < 0.05 is indicated by * and *p* < 0.01 is indicated by **.

**Table 5:** Effect of fall 2017 treatments on percent overwinter survival, number of frames with brood or adult bees and number of *N. ceranae* spores per bee in the spring 2018 for colonies treated with the antibiotic fumagillin, prebiotics eugenol, naringenin or chitosan, or probiotic Protexin^®^. Means are from 3 colonies for positive control, fumagillin S, eugenol P, chitosan S and chitosan P, 4 colonies for eugenol S and naringenin S, 5 colonies for negative control, Protexin^®^ S and naringenin P, and 6 colonies for Protexin^®^ P.

Treatment ^1^	% Survival ^2^	Frames	Frames	Spores/Bee ± SE^2^
		w/Brood ± SE^2^	w/Adults ± SE^2^	(Millions)
Positive Control	60	2.92 ± 2.2	3.17 ± 1.4	8.80 ± 2.22
Negative Control	83.4	4.45 ± 2.0	4.15 ± 1.6	1.90 ± 0.51
Fumagillin S	50	4.75 ± 0.8	6.42 ± 0.8	0.16 ± 0.16
Eugenol S	80	5.19 ± 1.6	5.44 ± 1.5	2.01 ± 0.91
Eugenol P	75	5.42 ± 1.0	6.00 ± 1.3	6.95 ± 6.77
Protexin S	100	5.60 ± 1.4	6.05 ± 1.6	1.38 ± 0.57
Protexin P	100	2.71 ± 1.1	3.54 ± 0.9	7.27 ± 3.05
Naringenin S	66.6	3.25 ± 0.7	3.44 ± 0.8	1.37 ± 0.67
Naringenin P	100	5.10 ± 1.7	5.10 ± 1.1	4.09 ± 1.71
Chitosan S	50	3.67 ± 1.5	4.00 ± 1.8	4.17 ± 2.80
Chitosan P	50	5.83 ± 3.1	5.08 ± 2.1	0.68 ± 0.59

^1^ Treatments applied in a protein patty (P) or sucrose syrup (S). In spring 2017, non-treated positive control and treated colonies started with natural *N. ceranae* infection, and non-treated negative control colonies started with no detectable *N. ceranae*. ^2^ No significant differences between treatments at *p* < 0.05 were detected based on analyses of variance.

**Table 6:** Effect of spring 2018 treatments on *N. ceranae* spore counts per bee one–two days pre-treatment and three weeks post-treatment with the prebiotic naringenin or probiotic Protexin^®^. Coefficients indicate how the log of spores per bee changed relative to the positive control. Means are from 100 bees in 33–34 colonies per treatment.

Treatment ^1^	Spores/Bee	Spores/Bee	% Change	Coefficient ^2^
	Pre-Treatment	Post-Treatment	SPORES/BEE	
	(Millions) ± SE	(Millions) ±SE		
Positive Control	14.19 ± 1.22	3.00 ± 0.35	−78.8	14.91
Protexin^®^ P	15.40 ± 1.20	2.04 ± 0.17	−86.7	−0.33 *
Naringenin P	15.60 ± 1.04	1.98 ± 0.17	−87.3	−0.42 *

^1^ Treatments applied in a protein patty (P)). Non-treated positive control and treated colonies started spring 2018 with natural *N. ceranae* infection. ^2^ Significance at *p* < 0.05 is indicated by *.

**Table 7:** Effect of spring 2018 treatments on number of frames containing brood per colony one–two days pre-treatment and nine weeks post-treatment with the prebiotic naringenin or probiotic Protexin^®^. Coefficients indicate how the number of frames with brood change changed relative to the positive control. Means are from 33–34 colonies per treatment.

Treatment ^1^	Frames w/Brood	Frames w/Brood	% Change	Coefficient ^2^
		Pre-Treat. ± SE	Post-Treat. ± SE	no. Frames
Positive Control	9.68 ± 0.35	11.30 ± 0.52	16.7	2.43
Protexin^®^ P	9.82 ± 0.34	10.70 ± 0.69	9	0.05
Naringenin P	9.93 ± 0.52	12.10 ± 0.70	21.8	0.06

^1^ Treatments applied in a protein patty (P). Non-treated positive control and treated colonies started spring 2018 with natural *N. ceranae* infection. ^2^ No significant differences between treatments at *p* < 0.05 were detected.

**Table 8:** Effect of spring 2018 treatments on number of frames covered with adult bees per colony one–two days pre-treatment and nine weeks post-treatment with the prebiotic naringenin or probiotic Protexin^®^. Coefficients indicate how the number of frames with adult bees changed relative to the positive control. Means are from 33–34 colonies per treatment.

Treatment ^1^	Frames w/Adults	Frames w/Adults	% Change	Coefficient ^2^
	Pre-Treat. ± SE	Post-Treat. ± SE	no. Frames	
Positive Control	13.1 ± 0.52	16.8 ± 0.87	28.2	2.82
Protexin^®^ P	12.3 ± 0.51	19.1 ± 1.03	55.3	0.13 *
Naringenin P	12.2 ± 1.04	18.9 ± 1.22	54.9	0.12 *

^1^ Treatments applied in a protein patty (P). Non-treated positive control and treated colonies started spring 2018 with natural *N. ceranae* infection. ^2^ Significance at *p* < 0.05 is indicated by *.

**Table 9:** Effect of fall 2018 treatments on *N. ceranae* spore counts per bee one-two days pre-treatment and three weeks post-treatment with the prebiotic naringenin or probiotic Protexin^®^. Coefficients indicate how the log of spores per bee changed relative to the positive control. Means are from 100 bees in 33–34 colonies per treatment.

Treatment ^1^	Spores/Bee	Spores/Bee	% Change	Coefficient ^2^
		Pre-Treatment	Post-Treatment	Spores/Bee
		(Millions) ± SE	(Millions) ± SE	
Positive Control	0.43 ± 0.17	0.37 ± 0.17	−13.9	12.82
Protexin^®^ S	0.49 ± 0.18	0.60 ± 0.17	22.4	−0.32
Naringenin S	0.47 ± 0.17	0.88 ± 0.17	87.2	−0.42

^1^ Treatments applied in a sucrose syrup (S). Non-treated positive control and treated colonies started spring 2018 with natural *N. ceranae* infection. ^2^ No significant differences between treatments at *p* < 0.05 were detected.

**Table 10:** Effect of fall 2018 treatments on percent overwinter survival, number of frames with brood or adult bees, and number of *N. ceranae* spores per bee in the spring 2019 for colonies treated with the prebiotic naringenin or probiotic Protexin^®^. Means are from 33 colonies for positive control and naringenin S and 34 colonies for Protexin^®^ S.

Treatment ^1^	% Survival ^2^	Frames	Frames	Spores/Bee± SE
		w/Brood ± SE ^2^	w/Adults ± SE ^2^	(Millions) ^2^
Positive Control	66.6 ^a^	4.5 ± 0.5 ^a^	5.4 ± 0.7 ^b^	10.6 ± 1.4 ^a^
Protexin^®^ S	79.4 ^a^	6.1 ± 0.6 ^a^	9.2 ± 1.1 ^a^	5.3 ± 0.9 ^b^
Naringenin S	69.7 ^a^	5.8 ± 0.6 ^a^	8.1 ± 0.9 ^a^	6.5 ± 1.3 ^b^

^1^ Treatments applied in a sucrose syrup (S). Non-treated positive control and treated colonies started spring 2018 with natural *N. ceranae* infection. ^2^ Different letters indicate significant differences of means at *p* < 0.05 based on Fisher’s exact tests for survival and ANOVA and LSD tests of log-transformed data for the other variables. Non-transformed values are presented.

## Data Availability

The data analyzed for the study are available from the corresponding author on reasonable request.
